# Characterizing myths of sexual aggression in the young population in Spain

**DOI:** 10.1186/s12889-024-19430-9

**Published:** 2024-07-19

**Authors:** Belén Sanz Barbero, Carmen Vives-Cases, Laura Vall-llosera Casanovas, Laura Serra Saurina, María Carme Saurina Canals, Gemma Renart Vicens

**Affiliations:** 1 National School of Public Health, Instituto de Salud Carlos III, Madrid, Spain; 2 Consortium for Biomedical Research in Epidemiology and Public Health (CIBERESP), C. de Sinesio Delgado, 4, Madrid, 28029 Spain; 3 Public Health Research Group, Department of Community Nursing, Preventive Medicine and Public Health and History of Science, Alicante University, Alicante, Spain; 4 Research Group on Statistics, Econometrics and Health (GRECS), University of Girona, Girona, Spain

**Keywords:** Myths of sexual aggression, Sexual violence, Violence against women, Youth, Spain

## Abstract

**Background:**

Myths of sexual aggression have a negative influence in aggressive behavior against women, in the institutional approaches to sexual violence and in how women cope with it. The objective of this study is to describe acceptance of myths of sexual aggression in young women and men residing in Spain.

**Method:**

Cross-sectional study carried out online with 2,515 women (50.2%) and men (49.8%) ages 18–35 in Spain in 2020. Information on myths was collected using the Acceptance of Modern Myths About Sexual Aggression Scale (AMMSA). We described the myths most prevalent among women and men. The variables associated with myths were identified using multiple regression. The regression models were adjusted by sociodemographic and sexual orientation variables.

**Results:**

The average AMMSA values were higher among men [mean: 3.11; standard deviation (sd):1.23] than among women (mean 2.49 sd:1.11). In both sexes, the myths with greater acceptance showed the presence of patriarchal gender roles in sexual contacts. Men were more likely than women to accept myths that question allegations and severity of violence. Having a higher level of educational studies (β -0.350 sd: 0.046) was associated with lower average AMMSA values. Being born in Latin America (β 0.047 sd: 0.063) was associated with higher average AMMSA values. Among heterosexual men, AMMSA values were greater than among gay and bisexual men. Among women, there was no difference in average AMMSA values based on sexual orientation.

**Conclusions:**

Myths persist during youth that question and trivialize sexual violence against women. It is necessary to implement strategies that reduce these myths, particularly in heterosexual men, in those of foreign-born origin and among those with low levels of education.

**Supplementary Information:**

The online version contains supplementary material available at 10.1186/s12889-024-19430-9.

## Background

Sexual violence (SV) encompasses any act, attempt or action against a person’s sexuality using coercion, regardless of their relationship to the victim [1]. SV is a violation of human rights that threaten freedom, dignity, security, and the right to life. They primarily affect women and girls and are perpetrated almost exclusively by men [2].

Prevention of SV is a public health priority, due to its magnitude, negative impact on mental and physical health and structural nature [3]. It is estimated that 11% of women in the European Union have suffered SV at some point since 15 years of age [4]. In Spain in 2019, 13.7% of women over age 16 reported having suffered SV at some point in life, and SV was more frequent among women ages 18–24 (19.1%) [5].

Despite its high prevalence, seeking help in situations of violence against women is scarce. Recent studies carried out in Spain show that young adult women who suffer intimate partner sexual violence are the least likely to report these events to formal services (social services, health services, community services) [6] Reporting of sexual crimes to police is still infrequent. In the EU-28 it is estimated that 15% of women that experience SV declare this aggression to the police [4]. In Spain, an estimated 8% of women that have suffered from SV outside of an intimate partnership reported these incidents [5]. There are also a scarce number of convictions for sexual crimes among cases [7, 8]. Studies link this low frequency of reports of sexual violence and small number of convictions to the social acceptance of rape myths [9, 10].

Rape myths are a set of beliefs that create a cognitive schema guiding the interpretation of information and events that occur in a sexual assault. The concept was originally defined by Burt [11] and refined by Bohner in 1998, and refers to a set of “*descriptive or prescriptive beliefs about rape (that is*, *about its causes*, *context*, *consequences*, *perpetrators*, *victims*, *and their interaction) that serve to deny*, *belittle*, *or justify sexual violence by men against women*.” [12]. Research on rape myths has traditionally focused on sexual assaults of extreme violence [13], leading to estimates possibly biased by social desirability.

Subsequently, Gerger and collaborators [14] developed the Scale of Acceptance of Modern Myths about Sexual Aggression (AMMSA), which gathers perceptions about a wider range of sexual assaults [15]. AMMSA has been validated in Spain by Megias and collaborators among students from the University of Granada (Spain), proving to be a useful tool for the analysis of social perception regarding sexual assaults [15].

Studies on Myths of Sexual Aggression suggest they have a negative influence on aggressive behavior [16, 17] institutional approaches to sexual violence (VS) [18], recognition of VS [19], and how women cope with it [20]. Given the high prevalence of VS among young women in Spain, the aim of this study is to characterize the level of acceptance of myths about sexual aggression among young men and women residing in Spain, in the year 2020.

## Methodology

### Population and sample

Cross-sectional study based on the online survey “Sexual Violence Among Young People”, carried out with women and men ages 18–35 residing in Spain (see additional file questionnaire). The sample size calculation was carried out taking into account recent data on the prevalence of SV in Spain, obtained from the Macrosurvey on Violence Against Women [5], corrected for sex. Data on the Spanish population ages 18–35 were obtained from the National Statistics Institute [21]. A minimum sample size was calculated of 2,500 questionnaires, to ensure a sample error of ± 5%, considering a 95% confidence level and prevalence estimates with a precision of (+/-) 0.9. In order to ensure that the sample was representative of the young population residing in Spain, quotas for sex, age, and autonomous community were applied. The participating individuals were selected from an online panel of 138,393 adults aged 16 and older. The panel was representative of the non-institutionalized civilian Spanish population. Participants were recruited via email and those who accepted received a link to respond to the survey. After conducting a pilot study, a database with 2,515 records collected between September 30 and October 28, 2020, was obtained. The response rate was 63.2%. Participation required the signing of a signed informed consent. The study was conducted according to the guidelines of the Declaration of Helsinki and was approved by the Ethics Committee of the University of Alicante (ref. UA-2020-07-07).

### Dependent variable

The measurement of the acceptance of myths about sexual aggression was conducted using the Acceptance of Modern Myths about Sexual Aggression Scale (AMMSA) [15], which consists of 30 items. These items form a single construct that covers the following categories: (a) denial of the problem; (b) questioning the intentions of the women who report; (c) critical position regarding the support provided to women by public policies; (d) naturalization of the lack of male sexual control; (e) beliefs that blame the victim, the circumstances and justify the aggressor’s behavior. The items are straightforward and are answered on a 7-point Likert scale (range: 1–7). Higher values indicate greater acceptance of myths about sexual aggression. In our study, the scale showed high internal consistency (α = 0.95), with values similar to those obtained in its validation (α = 0.91) [15].

#### Covariables

Based on previously published studies [16, 22], we included the following covariables: sex (male/female), age, country of birth (Spain/Latin America), highest level of education completed (up to secondary/higher education); sexual orientation (gay/lesbian/bisexual/heterosexual); paid work activity, last 12 months (yes/no); relationship status, last 12 months (yes/no).

### Analysis

First, we described the sample, globally and stratified by sex, according to the abovementioned covariables. Later we described the average AMMSA values by covariable categories. The differences in average AMMSA values between categories of covariables was carried out using the Student t-test for dichotomous variables and using the ANOVA in the case of variables with more categories. Later we described the average values of the items with greater or lesser acceptance, in the total sample and stratified by sex, as well as the items with the greatest and least differences between women and men. The difference in the average values for each item between men and women (difference = men’s average - women’s average) was analyzed using the ANOVA test. Finally, we analyzed the relationship between the AMMSA factor and the covariables using multiple regression analysis. In a prior step, analyses of residuals were carried out in order to verify that they were homoscedastic and that there was no multicollinearity. We explored interactions between the variable sex and the rest of the included covariables in the model and identified an interaction between sex and sexual orientation.

## Results

The database analyzed included 2,515 entries, of which 50.2% were women and 49.8% were men. Table 1 describes the characteristics of the total sample and the sample stratified by sex.

**Table 1:** Description of the study sample on myths about sexual violence. Young people 18–35 years old. Spain, 2020

Variables	Sex						Total sample	
	Women			Men				
	n (*n* = 1262)	% (50.2%)		n (*n* = 1253)	% (49.8%)		*n* (2515)	%
**Age**								
18–24 years	438	34.7		432	34.5		870	34.6
25–29 years	354	28.1		352	28.1		706	28.1
30–35 years	470	37.2		469	37.4		939	37.3
**Highest level of education completed**								
Primary and lower	16	1.3		17	1.4		33	1.3
Secundary	330	26.1		408	32.6		738	29.3
Higher education	903	71.6		816	65.1		1719	68.3
Not answer	13	1.0		12	1.0		25	1.0
**Sexual orientation**								
Lesbian	26	2.1		-	-		26	1.0
Gay	-	-		122	9.7		122	4.9
Bisexual	292	23.1		74	5.9		366	14.6
Heterosexual	925	73.3		950	75.8		1875	74.6
Not answer	19	1.5		107	8.5		126	5.0
**Paid work activity**, **last 12 months**								
Non	284	22.5		252	20.1		536	21.3
Yes	966	76.5		995	79.4		1961	78.0
Not answer	12	1.0		6	0.5		18	0.7
**Country of birth**								
Latin America	178	14.1		129	10.3		307	12.2
Spain	1084	85.9		1124	89.7		2208	87.8
**Relationship status**, **last 12 months**								
Non	351	27.8		456	36.4		807	32.1
Yes	886	70.2		770	61.5		1656	65.8
Not answer	25	2.0		27	2.2		52	2.1

Table 2 shows the average AMMSA values based on the covariable categories. AMMSA had an average value per item of 2.80 and a standard deviation (sd) of 1.21. The average AMMSA values were significantly greater in men [3.11(sd:1.23)], in those with a level of education up to primary studies [3.51(sd:1.26)], in heterosexual people [2.91(sd:1.20)], those born in Latin America [3.11(sd:1.29)], people not actively employed [2.94(sd:1.27)] and those not currently in an intimate relationship [2.91 (sd:1.22)] (Table 2).

**Table 2:** Mean values per item of the modern myths about sexual assault (AMMSA) scale and standard deviation according to sociodemographic characteristics. Young people 18–35 years old. Spain, 2020

Variables	Mean	Standar deviatio	*p*
Sex			
Woman	2.49	1.11	< 0.001
Man	3.11	1.23	
**Age**			< 0.001
18–24 years	2.83	1.24	
25–29 years	2.66	1.17	
30–35 years	2.88	1.21	
**Highest level of education completed**			
Primary and lower	3.51	1.26	< 0.001
Secundary	3.17	1.20	
Higher education	2.62	1.17	
**Sexual orientation**			< 0.001
Lesbian	2.29	1.16	
Gay	2.48	1.17	
Bixesual	2.40	1.14	
Heterosexual	2.91	1.20	
**Paid work activity. last 12 months**			0.003
Non	2.94	1.27	
Yes	2.76	1.19	
**Country of birth**			< 0.001
Latin America	3.11	1.28	
Spain	2.76	1.19	
**Relationship status. last 12 months**			0.002
Non	2.91	1.22	
Yes	2.75	1.20	
**Total**	**2.80**	**1.21**	

P values obtained with the Student t test for dichotomous variables and by the ANOVA test for variables with more than two categories

Table 3 shows the AMMSA scale items that obtained the most extreme scores. The average score for all of the items of the AMMSA scale can be found in the additional material [see Additional file 1]. For all of the items the average score was significantly greater in men than in women. Among both sexes the items that obtained the higher scores were item-19, which criticizes the opportunistic use of SV in politics and the media, and item-1, which shows traditional roles in sexual contacts. Next, there are the items critical of the support women who are victims of sexual aggression receive (item-25).

**Table 3:** Items from the modern myths about sexual assault (AMMSA) scale with upper and lower mean values. Total data and stratified by sex. Young people 18–35 years old. Spain. 2020

	Items from AMMSA scale with upper values	Total (*n* = 2515)			Women (*n* = 1262)			Men (*n* = 1253)				*p*
		M	sd		M	sd		M	sd			
Item_19	When politicians deal with the topic of rape they do so mainly because this topic is likely to attract the attention of the media.	4.42	2.07		4.26	2.07		4.57	2.07			< 0.001
Item_1	When it comes to sexual contacts women expect men to take the lead.	4.08	1.91		3.83	1.91		4.34	1.89			< 0.001
Item_25	Although the victims of armed robbery have to fear for their lives they receive far less psychological support than do rape victims.	3.66	2.05		3.44	2.06		3.87	2.03			< 0.001
Item_7	After a rape woman nowadays receive ample support.	3.60	2.07		3.28	2.03		3.93	2.06			< 0.001
Item_28	Nowadays the victims of sexual violence receive sufficient help in the form of women’s shelters therapy offers and support groups.	3.38	1.98		3.09	1.97		3.67	1.94			< 0.001
Item_5	Interpreting harmless gestures as “sexual harassment” is a popular weapon in the battle of the sexes.	3.21	2.07		2.73	2.00		3.69	2.01			< 0.001
	**Items from AMMSA scale with lower values**											
Item_11	Any woman who is careless enough to walk through “dark alleys” at night is partly to be blamed if she is raped.	1.57	1.33		1.36	1.12		1.77	1.49			< 0.001
Item_12	When a woman starts a relationship with a man she must be aware that the man will assert his right to have sex.	1.90	1.61		1.66	1.48		2.14	1.70			< 0.001
Item_30	Nowadays men who really sexually assault women are punished justly.	2.11	1.63		1.70	1.32		2.52	1.80			< 0.001
Item_17	When a man urges his female partner to have sex this cannot be called rape.	2.12	1.71		1.77	1.55		2.48	1.80			< 0.001
Item_3	A lot of women strongly complain about sexual infringements for no real reason just to appear emancipated.	2.29	1.71		1.89	1.50		2.69	1.82			< 0.001
Item_21	A man’s sexuality functions like a steam boiler, when the pressure gets too high he has to “let off steam.”	2.37	1.86		2.18	1.81		2.56	1.88			< 0.001

M: mean; sd: standard deviation. P values obtained through the ANOVA test

The items that had lower scores in both sexes were those that directly blame women or their circumstances (item-11), items that consider the penalties for the aggressors to be appropriate (item-30) and items that question rape in a relationship (item-17).

The greatest differences between women and men, with higher values in men (Table 4) were for items that refer to the incorrect interpretation of men’s behavior (item-5), items that refer to women’s exaggeration of SV (items-27, 16, 23) and the use of false accusations of SV to obtain legal benefits. Also observed among men was a more critical position in terms of the severity of SV in society (item-29). The highest degree of consensus between women and men (Table 4) was found in items with low acceptance among both sexes, such as those that exonerate the aggressor (Table 4).

**Table 4:** Items from the modern myths about sexual assault (AMMSA) scale with major and minor differences between women and men. Young people 18–35 years old. Spain. 2020

	Items from AMMSA scale with major differences between men and women	Total (*n* = 2515)			Women (*n* = 1262)			Men (*n* = 1253)			Mean difference		*p*
		M	sd		M	sd		M	sd		d		
Item_5	Interpreting harmless gestures as “sexual harassment” is a popular weapon in the battle of the sexes.	3.21	2.07		2.73	2.00		3.69	2.01		0.96		< 0.001
Item_27	Many women tend to misinterpret a well-meant gesture as a “sexual assault”.	2.70	1.84		2.23	1.68		3.19	1.87		0.96		< 0.001
Item_4	To get custody for their children. women often falsely accuse their ex-husband of a tendency towards sexual violence.	3.04	1.99		2.58	1.87		3.50	1.99		0.92		< 0.001
Item_16	Many women tend to exaggerate the problem of sexist violence.	2.65	1.96		2.21	1.79		3.09	2.03		0.88		< 0.001
Item_23	The discussion about sexual harassment on the job has mainly resulted in many a harmless behavior being misinterpreted as harassment.	2.96	1.92		2.52	1.81		3.39	1.94		0.87		< 0.001
Item_29	Instead of worrying about alleged victims of sexual violence society should rather attend to more urgent problems. such as environmental destruction.	2.35	1.63		1.92	1.37		2.78	1.75		0.87		< 0.001
	**Items from AMMSA scale with minor differences between men and women**												
Item_8	Nowadays a large proportion of rapes is partly caused by the depiction of sexuality in the media as this raises the sex drive of potential perpetrators.	2.78	1.98		2.68	2.00		2.89	1.95		0.21		0.004
Item_20	When defining “marital rape” there is no clear-cut distinction between normal conjugal intercourse and rape.	2.97	2.06		2.85	2.12		3.08	1.99		0.23		0.001
Item_19	When politicians deal with the topic of rape they do so mainly because this topic is likely to attract the attention of the media.	4.42	2.07		4.26	2.07		4.57	2.07		0.31		< 0.001
Item_14	Because the fascination caused by sex is disproportionately large. our society’s sensitivity to crimes in this area is disproportionate as well.	2.64	1.87		2.46	1.88		2.82	1.84		0.36		< 0.001
Item_24	In dating situations, the general expectation is that the woman “hits the brakes” and the man “pushes ahead”.	2.60	1.83		2.41	1.83		2.79	1.82		0.37		< 0.001
Item_21	A man’s sexuality functions like a steam boiler, when the pressure gets too high he has to “let off steam”.	2.37	1.86		2.18	1.81		**2.56**	1.88		0.38		< 0.001

M: mean; sd: standard deviation; d: mean differences (Mman-Mwoman)

P values obtained through the ANOVA test

Table 5 shows the variables associated with acceptance of myths of sexual aggression. The average acceptance of myths of sexual aggression was lower in people with a higher level of education - reference: up to secondary studies- (β:-0.350 sd: 0.046). The average value of AMMSA was higher in people born in Latin America - reference: Spain - (β:0.250 sd: 0.063) (Table 5).

**Table 5:** Variables associated with the mean values of the modern myths about sexual assault (AMMSA) scale. Multiple linear regression. Young people 18–35 years old. Spain. 2020

Variables	B	SE B	*p*-value
**Sex** (Reference: Man)			
Woman	-0.262	0.095	0.006
**Aged (years)**	0.011	0.005	0.017
**Highest level of education completed** (Reference: Secundary and lower)			
Higher education	-0.350	0.046	< 0.001
**Sexual Orientation** (Reference: Lesbian/Gay/Bisexual)			
Heterosexual	0.425	0.067	< 0.001
**Paid work activity. last 12 months** (Reference: Non)			
Yes	-0.024	0.054	0.656
**Country of birth** (Reference: Spain)			
Latin America	0.250	0.063	< 0.001
**Relationship status. last 12 months** (Reference: Non)			
Yes	-0.066	0.045	0.140
**Interaction: Sexual Orientation x Sex**	-0.345	0.104	< 0.001
**Constant**	-0.152	0.135	0.259

**Fig. 1:**
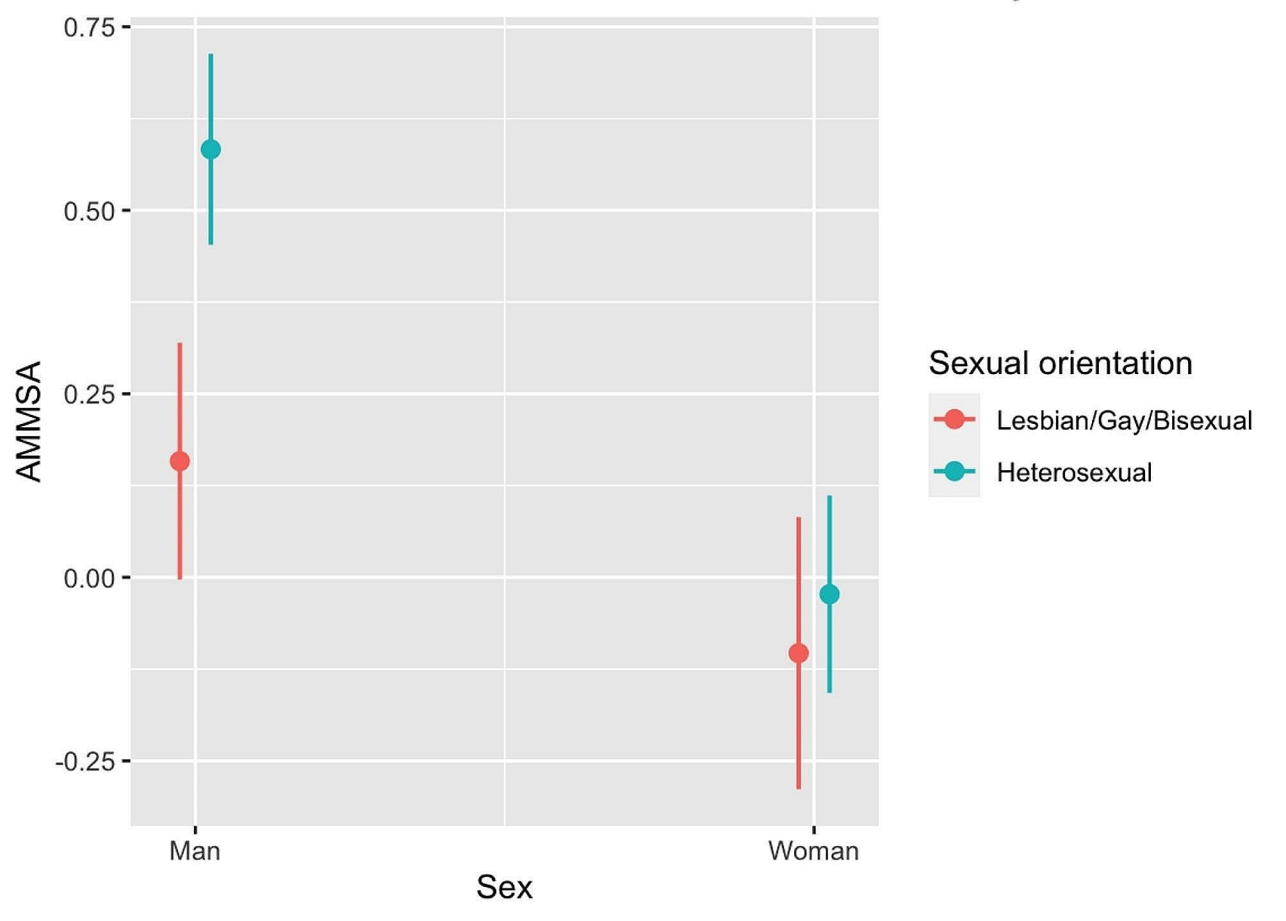
Predictions of the AMMSA factor according to sex and sexual orientation. Young people 18–35 years old. Spain. 2020

An interaction was identified between sexual orientation and sex (Fig. 1). In heterosexual men average AMMSA values were higher than in gaysexual/bisexual men. For women there was no difference in the average AMMSA values by sexual orientation.

## Discusion

The acceptance of myths of sexual aggression was greater in men than in women. Among both sexes, the myths with greater acceptance refer to the presence of patriarchal gender roles in sexual contacts and to the opportunistic use of SV in politics and in the media. There are myths that maintain a critical stance towards institutional support for victims. Men had greater acceptance than women of myths that question reporting of SV or trivialize the severity of SV. Being a heterosexual man, lacking a higher level of education and being born in Latin America was associated with a greater acceptance of myths of sexual aggression.

Average acceptance of myths of sexual aggression found in this work was less than what was identified by Mejías and colleagues in 2011 [15] with a sample of university students. This result could suggest a decrease in the acceptance of myths of sexual aggression in the past decade, primarily among women. This fact could be related to a greater level of awareness and more critical social stance towards SV in Spain, in part prompted by the influence of cases of SV with a high level of media attention among the young population and by worldwide women’s social movements such as #MeToo2017 and #YoSiTeCreo-2018 [23].

Despite this possible decrease in the acceptance of myths of sexual aggression, in the young population, myths persist that reflect patriarchal gender roles in sexual contacts and that question SV and the institutional response to the problem. This negation, trivialization of SV and questioning of reporting, observed primarily among men, has important implications both in terms of victim’s request for help [10, 13] and in the victim’s recuperation of wellbeing [24] as well as in the response of SV support services [9, 25]. Prior studies show that the probability of a woman seeking help decreases when she perceives that her testimony could be questioned [10]. At the same time, other studies have identified an association between the acceptance of myths of sexual aggression by professionals in the police and judicial services and secondary victimization of women who suffer from SV - called “judicial rape” [26]. This secondary victimization related to the lack of credibility in women’s testimonies has been associated with the withdrawal of complaints [18], which can also serve to strengthen the myth of false complaints.

In this study, myths that had a lower level of acceptance among both sexes were those that blame the victim and that do not recognize SV in a couple relationship. This result does not agree with what has been observed in qualitative studies of young people, carried out in England and Wales [27] and in United States of America [28], in which discourses have emerged that blame the victims [27, 28]. The distinct methodological approach, as well as the possible social desirability bias present in our work related to the more extreme items could explain these differences [14].

There was also a greater acceptance in men than in women of myths that question situations of assault. This result could be a reflection of men’s normalization of sexual assault, an important point given the high prevalence of assault both in Spain and in the European context [4, 5, 29] and its impact on women’s health and wellbeing [30]. In this sense, the new Organic Law on Sexual Liberty [31] has defined street harassment of a sexual nature as a crime.

Our results show an associate between the acceptance of myths of sexual aggression and the sociodemographic characteristics of young people. Specifically, we observed a greater acceptance of myths of sexual aggression in those with a lower level of education, those born in Latin America and in heterosexual men. Although information on the association between myths of sexual aggression and sociodemographic variables of young people is scarce [16], the acceptance of myths of sexual aggression has shown a high correlation with scales that measure social constructs of hostility towards women, such as ambivalent sexism [32]. Having a low education level has been associated with myths acceptance in our work, and it has also been associated with higher average levels of ambivalent sexism in both sexes [33]. In terms of place of origin, to our knowledge, there are no prior studies that analyze the association between migratory status and acceptance of myths of sexual aggression. Although scarce, there are studies conducted in England and Wales that identify a positive association between belonging to an ethnic minority and acceptance of these myths [27]. It is necessary to carry out more studies that analyze the reasons underlying these associations and avoid the stigmatization of social groups in which different axes of inequality intersect.

One novel finding of our study is the association between sexual orientation and acceptance of myths of sexual aggression, an association modified by sex. Heterosexual men showed a greater acceptance of myths of sexual aggression than gay/bisexual men. It is possible that ambivalent sexism, primarily in the dimension of hostile sexism, which is more present among heterosexual men than among gay/bisexual men, could partly explain this association [34, 35]. In our work, there was no association between sexual orientation and acceptance of myths of sexual aggression in women. Also, no association between sexual orientation and hostile sexism has been identified in women [36].

This study should be interpreted taking into account its limitations and strengths. Although the sample in this study was not randomly selected, the sample size and representativeness of the sample were designed to maximize external validity. In terms of the scale used -AMMSA-, it is possible that the items that refer to more serious behaviors could be subject to social desirability bias. We were not able to analyze the categories that make up the sexual orientation variable independently in the regression model due to the small number of cases in some categories. Due to the composition of the panel used, the immigrant population included in this work was exclusively of Latin American origin. Even though we were unable to extrapolate the results to other foreign origin groups, it provides information on an immigrant group from a single region of origin. Despite these limitations, our results provide new information on an understudied topic which is currently of great importance.

## Conclusions

Myths of sexual aggression persist among the young population in Spain and are more present among men than among women. Greater differences by sex were found for myths that question and trivialize SV suffered by women, and these were more accepted by men. Sociodemographic variables were associated with sexual aggression myths independently of sex, while the association between sexual orientation and these myths differed between men and women. It is necessary to implement public policies that reduce the acceptance of myths about sexual assaults in the Spanish population. These policies should have a comprehensive approach, incorporating the different axes of inequality that converge in the population.

### Electronic supplementary material

Below is the link to the electronic supplementary material.

Supplementary Material 1

Supplementary Material 2

## Data Availability

“The datasets and material-questionnaire- that have been produced during the current study are available from the corresponding author on reasonable request that guarantees their use according to the ethical procedures adopted in this project and participants’ informed consent documents content.”

## Abbreviations

AMMSA: Modern Myths About Sexual Aggression Scale
EU-28: European Union
sd: Standard Deviation
SV: Sexual Violence

## References

[1] Krug E, Dahlberg L, Mercy J, Zwi A, Lozano R. The world health report 2002: reducing risks, promoting healthy life. Geneve: World Health Organization.; 2002.

[2] Breiding MJ. Prevalence and characteristics of sexual violence, stalking, and intimate partner violence victimization—national intimate Partner and sexual violence Survey, United States, 2011. Morbidity Mortal Wkly Rep Surveillance Summaries (Washington DC: 2002). 2014;63(8):1. PMC4692457 25188037

[3] Women UN. Beijing Declaration and platform for action-Beijing + 5 political declaration and outcome. https://dspace.ceid.org.tr/xmlui/handle/1/1272 [consultado 27/11/2023] 1995.

[4] European Union Agency for Fundamental Rights. Violence Against Women Survey, main results. 2014 https://fra.europa.eu/sites/default/files/fra_uploads/fra-2014-vaw-survey-main-results-apr14_en.pdf. Accessed 15 Nov 2023.

[5] Ministerio de Igualdad. Delegación de Gobierno contra la Violencia de Género. Macroencuesta de Violencia contra las Mujeres 2019. 2019 https://violenciagenero.igualdad.gob.es/violenciaEnCifras/macroencuesta2015/pdf/Macroencuesta_2019_estudio_investigacion.pdf. Accessed 15 Nov 2023.

[6] Sanz-Barbero B, Briones-Vozmediano E, Otero-García L, Fernández-García C, Vives-Cases C. (2022). Spanish intimate partner violence survivors help-seeking strategies across the life span. J. Interpers. Violence. 2022;37(11–12):NP8651-NP8669. 10.1177/0886260520976213 33289463

[7] López Gutiérrez J, Sánchez Jiménez F, Herrera Sánchez D, Martínez Moreno F, Rubio García M, Gil Pérez MV, Santiago Orozco AM, Góme Martín MA. Informe sobre delitos contra la libertad e indemnidad sexual. Ministerio del Interior. 2021. https://www.interior.gob.es/opencms/pdf/prensa/balances-e-informes/2021/Informe-delitos-contra-la-libertad-e-indemnidad-sexual-2021.pdf Accessed 20 Jan 2024.

[8] Instituto Nacional de Estadística. Condenados por delitos sexuales según sexo, edad y nacionalidad 2021. https://www.ine.es/jaxiT3/Tabla.htm?t=28857&L=0. Accessed 20 Nov 2023.

[9] Sleath E, Bull R. Police perceptions of rape victims and the impact on case decision making: a systematic review. Aggress Violent Behav. 2017;34:102–12. 10.1016/j.avb.2017.02.003

[10] Egan R, Wilson JC. Rape Victims’ Attitudes to Rape Myth Acceptance. Psychiatry, Psychology and Law. 2012;19(3):345 – 57.

[11] Burt M. Cultural myths and supports for rape. J Pers Soc Psychol. 1980;38(2):217. 7373511 10.1037/0022-3514.38.2.217

[12] Bohner G, Vergewaltigungsmythen. Sozialpsychologische Untersuchungen über täterentlastende und opferfeindliche Überzeugungen Im Bereich Sexueller Gewalt [Rape myths: Social psychological studies of perpetrator-exonerating and victim-hostile beliefs in the area of sexual violence]. Landau, Germany: Verlag Empirische Pädagogik; 1998.

[13] Payne D, Lonsway K, Fitzgerald L. Rape myth acceptance: exploration of its structure and its measurement using theIllinois rape myth acceptance scale. J res Pers. 1999;33(1):27–68. 10.1006/jrpe.1998.2238

[14] Gerger H, Kley H, Bohner G, Siebler F. The acceptance of modern myths about sexual aggression scale: development and validation in German and English. Aggress Behav. 2007;33(5):422–40. 17683102 10.1002/ab.20195

[15] Megias JL, Romero-Sanchez M, Duran M, Moya M, Bohner G. Spanish validation of the Acceptance of Modern Myths about sexual aggression scale (AMMSA). Span J Psychol. 2011;14(2):912–25. 22059335 10.5209/rev_SJOP.2011.v14.n2.37

[16] Fernández-Fuertes AA, Fernández-Rouco N, Lázaro-Visa S, Gómez-Pérez E. Myths about sexual aggression, sexual assertiveness and sexual violence in adolescent romantic relationships. Int J Environ Res Public Health. 2020;17(23):8744. 33255546 10.3390/ijerph17238744 PMC7727844

[17] Bohner G, Siebler F, Schmelcher J. Social norms and the likelihood of raping: perceived rape myth acceptance of others affects men’s rape proclivity. Pers Soc Psychol Bull. 2006;32(3):286–97. 16455857 10.1177/0146167205280912

[18] Garza A, Franklin C. The effect of rape myth endorsement on police response to sexual assault survivors. Violence against Women. 2021;27(3–4):552–73. 32241227 10.1177/1077801220911460

[19] Lathan E, Koon-Magnin S, Selwyn C, Isaak H, Langhinrichsen-Rohling J. Rape myth acceptance and other barriers to formally reporting sexual assault among college students with and without sexual assault histories. J Interpers Violence. 2023;38(9–10):6773–97. 36421002 10.1177/08862605221137703

[20] Henry T, Franklin T, Franklin C. Facilitating sexual assault reporting on the College campus: the role of Procedural Justice in Bystander decisions to provide police referrals. Violence against Women. 2021;27(11):2066–91. 32954994 10.1177/1077801220954289

[21] Instituto Nacional de EstadísticaEstadística. Población residente por fecha, sexo y edad 2021 https://www.ine.es/jaxiT3/Tabla.htm?t=56934. Accessed 20 Nov 2023.

[22] Stephens T, Kamimura A, Yamawaki N, Bhattacharya H, Mo W, Birkholz R et al. Rape myth Acceptance among College students in the United States, Japan, and India. SAGE Open 2016;6(4).

[23] Nomamiukor F, Wisco B. Social Media’s Impact on Rape Myth Acceptance and Negative Affect in College Women: Examining the# MeToo and# HimToo Movement. Violence against Women. 2023:10778012231181045. 10.1177/10778012231181045 37345426

[24] Bernstein E, Kanefsky R, Cook M, Newins A. Acceptance of rape myths and psychological symptoms: the indirect effect of self-blame. J Am Coll Health. 2022:1–5. 10.1080/07448481.2022.2086005 35728073

[25] Goicolea I, Vives-Cases C, Castellanos-Torres E, Briones-Vozmediano E, Sanz-Barbero B. Disclosing Gender-Based Violence: A Qualitative Analysis of Professionals’ and Women’s Perspectives through a Discursive Approach. Int J Environ Res Public Health. 2022;19(22). 10.3390/ijerph192214683 PMC9690750 36429401

[26] Lees S. Judicial rape. In Women’s Studies International Forum (Vol. 16, No. 1, pp. 11–36). Pergamon.

[27] Hermolle M, Andrews SJ, Huang CS. Rape Stereotype Acceptance in the General Population of England and Wales. J Interpers Violence. 2022;37(23–24):NP23131–55. 35225066 10.1177/08862605221076162 PMC9679569

[28] Johnson NL, Corbett-Hone M, Gutekunst M, Wolf J. The grey zone of collegiate sexual regret: questionable consent and sexual victimisation. Cult Health Sex. 2021;23(2):159–75. 32141796 10.1080/13691058.2019.1696985

[29] Vall-Llosera L, Serra L, Canals CS, Sanz-Barbero B, Vives-Cases C, Lopez MJ, et al. Prevalence of sexual harassment among young spaniards before, during, and after the COVID-19 lockdown period in Spain. BMC Public Health. 2022;22(1):1888. 36221078 10.1186/s12889-022-14264-9 PMC9551249

[30] Chan DKSC, Lam SY, Cheung CB. Examining the Job-Related, psychological, and physical outcomes of workplace sexual harassment: a Meta-Analytic Review. Psychol Women Q. 2008;32:14. 10.1111/j.1471-6402.2008.00451.x

[31] Boletín Oficial del Estado. Ley Orgánica 10/2022, de 6 de septiembre, de garantía integral de la libertad sexual. https://www.boe.es/eli/es/lo/2022/09/06/10/con. Accessed 27 Nov 2023.

[32] Angelone D, Cantor N, Marcantonio T, Joppa M. Does sexism mediate the gender and rape myth acceptance relationship? Violence against Women. 2021;27(6–7):748–65. 32339090 10.1177/1077801220913632

[33] Garaigordobil M, Aliri J. Ambivalent sexism inventory: standardization and normative data in a sample of the Basque Country. Behav Phycho. 2013;21(1).

[34] Blumell LE, Rodriguez NS. Ambivalent Sexism and Gay men in the US and UK. Sex Cult. 2019;24(1):209–29. 10.1007/s12119-019-09635-1

[35] Cowie LJ, Greaves LM, Sibley C. Sexuality and sexism: differences in ambivalent sexism across gender and sexual identity. Pers Individ Differ. 2019;148:85–9. 10.1016/j.paid.2019.05.023

[36] Madrona-Bonastre R, Sanz-Barbero B, Pérez-Martínez V, Abiétar D, Sánchez-Martínez F, Forcadell-Díez L, et al. Sexismo Y violencia de pareja en adolescentes [Sexism and intimate partner violence in adolescents]. Gac Sanit. 2023;37:102221. 36113323 10.1016/j.gaceta.2022.02.007

