# Supplementary material for: Characterizing myths of sexual aggression in the young population in Spain
Source: BMC Public Health. 2024 Jul 19;24:1944. doi: 10.1186/s12889-024-19430-9 (PMC11265002; doi:10.1186/s12889-024-19430-9)
Supplement: Supplementary file 1 — Supplementary Material 1 [file 12889_2024_19430_MOESM1_ESM.docx]

Table A1: Differences by sex in the mean values per item of the AMMSA scale. Young people 18-35 years old. Spain

| Items AMMSA | Total | |  | Women | |  | Men | |  | Mean difference |  | p |
| --- | --- | --- | --- | --- | --- | --- | --- | --- | --- | --- | --- | --- |
|  | Mean | Sd |  | Mean | Sd |  | Mean | Sd |  | d |  |  |
| 1. When it comes to sexual contacts, women expect men to take the lead. | 4.08 | 1.91 |  | 3.83 | 1.91 |  | 4.34 | 1.89 |  | 0.51 |  | <0.001 |
| 2. Once a man and a woman have started "making out", a woman's misgivings against sex will automatically disappear. | 2.76 | 1.67 |  | 2.46 | 1.56 |  | 3.05 | 1.72 |  | 0.59 |  | <0.001 |
| 3. A lot of women strongly complain about sexual infringements for no real reason, just to appear emancipated. | 2.29 | 1.71 |  | 1.89 | 1.50 |  | 2.69 | 1.82 |  | 0.80 |  | <0.001 |
| 4. To get custody for their children, women often falsely accuse their ex-husband of a  tendency towards sexual violence. | 3.04 | 1.99 |  | 2.58 | 1.87 |  | 3.50 | 1.99 |  | 0.92 |  | <0.001 |
| 5. Interpreting harmless gestures as "sexual harassment" is a popular weapon in the battle of the sexes. | 3.21 | 2.07 |  | 2.73 | 2.00 |  | 3.69 | 2.01 |  | 0.96 |  | <0.001 |
| 6. It is a biological necessity for men to release sexual pressure from time to time. | 3.10 | 2.13 |  | 2.79 | 2.13 |  | 3.41 | 2.09 |  | 0.62 |  | <0.001 |
| 7. After a rape, women nowadays receive ample support. | 3.60 | 2.07 |  | 3.28 | 2.03 |  | 3.93 | 2.06 |  | 0.65 |  | <0.001 |
| 8. Nowadays, a large proportion of rapes is partly caused by the depiction of sexuality in the media as this raises the sex drive of potential perpetrators. | 2.78 | 1.98 |  | 2.68 | 2.00 |  | 2.89 | 1.95 |  | 0.21 |  | 0.004 |
| 9. If a woman invites a man to her home for a cup of coffee after a night out this means that she wants to have sex. | 2.53 | 1.83 |  | 2.14 | 1.69 |  | 2.91 | 1.89 |  | 0.77 |  | <0.001 |
| 10. As long as they don’t go too far, suggestive remarks and allusions simply tell a woman that she is attractive. | 2.67 | 1.81 |  | 2.27 | 1.70 |  | 3.08 | 1.84 |  | 0.81 |  | <0.001 |
| 11. Any woman who is careless enough to walk through “dark alleys” at night is partly to be blamed if she is raped. | 1.57 | 1.33 |  | 1.36 | 1.12 |  | 1.77 | 1.49 |  | 0.42 |  | <0.001 |
| 12. When a woman starts a relationship with a man, she must be aware that the man Will assert his right to have sex. | 1.90 | 1.61 |  | 1.66 | 1.48 |  | 2.14 | 1.70 |  | 0.48 |  | <0.001 |
| 13. Most women prefer to be praised for their looks rather than their intelligence. | 2.37 | 1.78 |  | 2.08 | 1.68 |  | 2.66 | 1.83 |  | 0.57 |  | <0.001 |
| 14. Because the fascination caused by sex is disproportionately large, our society’s sensitivity to crimes in this area is disproportionate as well. | 2.64 | 1.87 |  | 2.46 | 1.88 |  | 2.82 | 1.84 |  | 0.36 |  | <0.001 |
| 15. Women like to play coy. This does not mean that they do not want sex. | 3.26 | 2.14 |  | 2.92 | 2.14 |  | 3.61 | 2.08 |  | 0.69 |  | <0.001 |
| 16. Many women tend to exaggerate the problem of male violence. | 2.65 | 1.96 |  | 2.21 | 1.79 |  | 3.09 | 2.03 |  | 0.88 |  | <0.001 |
| 17. When a man urges his female partner to have sex, this cannot be called rape. | 2.12 | 1.71 |  | 1.77 | 1.55 |  | 2.48 | 1.80 |  | 0.71 |  | <0.001 |
| 18. When a single woman invites a single man to her flat she signals that she is not averse to having sex. | 2.62 | 1.84 |  | 2.29 | 1.75 |  | 2.96 | 1.86 |  | 0.68 |  | <0.001 |
| 19. When politicians deal with the topic of rape, they do so mainly because this topic is likely to attract the attention of the media. | 4.42 | 2.07 |  | 4.26 | 2.07 |  | 4.57 | 2.07 |  | 0.31 |  | <0.001 |
| 20. When defining "marital rape", there is no clear-cut distinction between normal conjugal intercourse and rape. | 2.97 | 2.06 |  | 2.85 | 2.12 |  | 3.08 | 1.99 |  | 0.23 |  | 0.001 |
| 21. A man’s sexuality functions like a steam boiler – when the pressure gets too high, he has to "let off steam". | 2.37 | 1.86 |  | 2.18 | 1.81 |  | 2.56 | 1.88 |  | 0.38 |  | <0.001 |
| 22. Women often accuse their husbands of marital rape just to retaliate for a failed  relationship. | 2.59 | 1.83 |  | 2.18 | 1.68 |  | 3.00 | 1.88 |  | 0.82 |  | <0.001 |
| 23. The discussion about sexual harassment on the job has mainly resulted in many a harmless behavior being misinterpreted as harassment. | 2.96 | 1.92 |  | 2.52 | 1.81 |  | 3.39 | 1.94 |  | 0.87 |  | <0.001 |
| 24. In dating situations the general expectation is that the woman "hits the brakes" and the man "pushes ahead". | 2.60 | 1.83 |  | 2.41 | 1.83 |  | 2.79 | 1.82 |  | 0.37 |  | <0.001 |
| 25. Although the victims of armed robbery have to fear for their lives, they receive far les psychological support than do rape victims. | 3.66 | 2.05 |  | 3.44 | 2.06 |  | 3.87 | 2.03 |  | 0.43 |  | <0.001 |
| 26. Alcohol is often the culprit when a man rapes a woman. | 2.72 | 1.97 |  | 2.51 | 1.94 |  | 2.94 | 1.98 |  | 0.43 |  | <0.001 |
| 27. Many women tend to misinterpret a well-meant gesture as a "sexual assault". | 2.70 | 1.84 |  | 2.23 | 1.68 |  | 3.19 | 1.87 |  | 0.96 |  | <0.001 |
| 28. Nowadays, the victims of sexual violence receive sufficient help in the form of women’s shelters, therapy offers, and support groups. | 3.38 | 1.98 |  | 3.09 | 1.97 |  | 3.67 | 1.94 |  | 0.58 |  | <0.001 |
| 29. Instead of worrying about alleged victims of sexual violence society should rather attend to more urgent problems, such as environmental destruction. | 2.35 | 1.63 |  | 1.92 | 1.37 |  | 2.78 | 1.75 |  | 0.87 |  | <0.001 |
| 30. Nowadays, men who really sexually assault women are punished justly. | 2.11 | 1.63 |  | 1.70 | 1.32 |  | 2.52 | 1.80 |  | 0.82 |  | <0.001 |
| total ammsa | 2.8 | 1.21 |  | 2.49 | 1.109 |  | 3.11 | 1.23 |  | 0.62 |  | <0.001 |

M:mean; Sd: standar deviation; d: mean differences (Mman-Mwoman)

P values obtained through the ANOVA test.
