# Supplementary material for: Characterizing myths of sexual aggression in the young population in Spain
Source: BMC Public Health. 2024 Jul 19;24:1944. doi: 10.1186/s12889-024-19430-9 (PMC11265002; doi:10.1186/s12889-024-19430-9)
Supplement: Supplementary file 2 — Supplementary Material 2 [file 12889_2024_19430_MOESM2_ESM.docx]

SEXUAL VIOLENCE SURVEY.

*Good morning/afternoon. The Carlos III Health Institute, the Public Health Agency of Barcelona, the University of Alicante, the Autonomous University of Madrid and the University of Girona, are conducting a study on aspects that are important in the lives of young people, specifically on sexual violence.*

*For this reason we ask for your collaboration and thank you in advance. Your contact has been selected randomly by random methods, using the panel of people interested in conducting surveys in which you are subscribed voluntarily. We guarantee the absolute anonymity and secrecy of your answers in strict compliance with the laws on statistical confidentiality and protection of personal data (include reference to legislation). Once the information has been recorded anonymously, the individual questionnaires are destroyed. If you agree to carry out the survey, please indicate this and start the questionnaire.*

**I agree to conduct the survey _______**

**P1 What year were you born? _____**

**P2. Sex**

- Man 1
- Woman 2
- No answer 9

**P3. Gender**

- Male 1
- Female 2
- Other (specified) 3
- No answer 9

**P4. Were you born outside of Spain??**

- No 1
- Yes (specified country) 2
- No answer 9

**P5. What is the highest level of education you have achieved?**

- Not completed primary education 1
- Completed primary education 2
- Completed secondary education 3
- Upper secondary education 4
- Others 5
- Do not know 8
- No answer 9

**P6.** **Have you ever had a paid job in your life?**

- Yes, currently 1
- Yes, previously 2
- No 3
- No answer 9

Filter on (P6=1,2)

**P7. Have you had a paid work last year?**

- Yes 1
- No 2
- No answer. 9

Filter off

**P8. Which of the following statements do you feel most identified with?**

- I am only attracted to women 1
- I am usually attracted to women but sometimes I am also attracted to men 2
- I am attracted to both women and men 3
- I am usually attracted to men but sometimes I am also attracted to women 4
- I am only attracted to men 5
- I am not attracted to women or men 6
- No answer 9

**P9. Have you ever had a romantic partner** (partner means any person with whom you have an affective-sexual relationship, whether or not you live together or have a legal relationship, and regardless of whether or not there is sexual intercourse, and regardless of the duration of the relationship)?

- Yes 1
- No 2
- No answer 9

**P10. Do you currently have a romantic partner?**

- Yes 1
- No 2
- No answer 9

Filter on (P10=1)

**P11. Do you live with your current partner?**

- Yes, we live in the same address 1
- Yes, but intermittently (seasons, weekends) 2
- No, we live in different addresses 3
- Others 4
- No answer 9

Filter off

Filter on (P9=1)

**P12.**  **Do you and/or your partner have minor children?**

- No 1
- Yes, they are children of both 2
- Yes, they are my children and of another couple *3*
- Yes, they are children of my partner with another partner 4
- No answer. 9

Filter off

**P13 “ACCEPTANCE OF MODERN MYTHS ABOUT SEXUAL AGGRESSION SCALE” (AMMSA).**

Below you will find a series of statements about men and women and their relationships. Please indicate the degree to which you agree or disagree with each statement using the following scale.

- Totally disagree 1

- ***Moderately*** Totally disagree 2

- Slightly disagree 3

- Neither disagree nor agree 4

- Slightly agree 5

- Moderately agree 6

- Totally agree 7

| 1. When it comes to sexual contacts, women expect men to take the lead. |  |
| --- | --- |
| 2. Once a man and a woman have started "making out", a woman's misgivings against sex will automatically disappear. |  |
| 3. A lot of women strongly complain about sexual infringements for no real reason, just to appear emancipated. |  |
| 4. To get custody for their children, women often falsely accuse their ex-husband of atendency towards sexual violence. |  |
| 5. Interpreting harmless gestures as "sexual harassment" is a popular weapon in the battle of the sexes. |  |
| 6. It is a biological necessity for men to release sexual pressure from time to time. |  |
| 7. After a rape, women nowadays receive ample support. |  |
| 8. Nowadays, a large proportion of rapes is partly caused by the depiction of sexuality in the media as this raises the sex drive of potential perpetrators. |  |
| 9. If a woman invites a man to her home for a cup of coffee after a night out this means that she wants to have sex. |  |
| 10. As long as they don’t go too far, suggestive remarks and allusions simply tell a woman that she is attractive. |  |
| 11. Any woman who is careless enough to walk through “dark alleys” at night is partly to be blamed if she is raped. |  |
| 12. When a woman starts a relationship with a man, she must be aware that the man Will assert his right to have sex. |  |
| 13. Most women prefer to be praised for their looks rather than their intelligence. |  |
| 14. Because the fascination caused by sex is disproportionately large, our society’s sensitivity to crimes in this area is disproportionate as well. |  |
| 15. Women like to play coy. This does not mean that they do not want sex. |  |
| 16. Many women tend to exaggerate the problem of male violence. |  |
| 17. When a man urges his female partner to have sex, this cannot be called rape. |  |
| 18. When a single woman invites a single man to her flat she signals that she is not averse to having sex. |  |
| 19. When politicians deal with the topic of rape, they do so mainly because this topic is likely to attract the attention of the media. |  |
| 20. When defining "marital rape", there is no clear-cut distinction between normal conjugal intercourse and rape. |  |
| 21. A man’s sexuality functions like a steam boiler – when the pressure gets too high, he has to "let off steam". |  |
| 22. Women often accuse their husbands of marital rape just to retaliate for a failed  relationship. |  |
| 23. The discussion about sexual harassment on the job has mainly resulted in many a harmless behavior being misinterpreted as harassment. |  |
| 24. In dating situations the general expectation is that the woman "hits the brakes" and the man "pushes ahead". |  |
| 25. Although the victims of armed robbery have to fear for their lives, they receive far les psychological support than do rape victims. |  |
| 26. Alcohol is often the culprit when a man rapes a woman. |  |
| 27. Many women tend to misinterpret a well-meant gesture as a "sexual assault". |  |
| 28. Nowadays, the victims of sexual violence receive sufficient help in the form of women’s shelters, therapy offers, and support groups. |  |
| 29. Instead of worrying about alleged victims of sexual violence society should rather attend to more urgent problems, such as environmental destruction. |  |
| 30. Nowadays, men who really sexually assault women are punished justly. |  |
